# Impact of bedaquiline resistance probability on treatment decision for rifampicin-resistant TB

**DOI:** 10.5588/ijtldopen.24.0362

**Published:** 2024-09-01

**Authors:** T.P.H. Trang, R. Kessels, T. Decroo, A. Van Rie

**Affiliations:** ^1^ Department of Family Medicine and Population Health, Faculty of Medicine and Health Sciences, University of Antwerp, Antwerp, Belgium;; ^2^ Division of Pharmacoepidemiology and Clinical Pharmacology, Utrecht Institute for Pharmaceutical Sciences, Utrecht University, Utrecht, The Netherlands;; ^3^ Department of Data Analytics and Digitalization, Maastricht University, Maastricht, The Netherlands;; ^4^ Department of Economics, University of Antwerp, Antwerp, Belgium;; ^5^ Department of Clinical Sciences, Institute of Tropical Medicine, Antwerp, Belgium.

**Keywords:** discrete choice experiment, clinical decision-making, treatment uncertainty

## Abstract

**BACKGROUND:**

Accurate diagnosis of bedaquiline (BDQ) resistance remains challenging. A Bayesian approach expresses this uncertainty as a probability of BDQ resistance (prBDQ^R^) with a 95% credible interval. We investigated how prBDQ^R^ information influences BDQ prescribing decisions.

**METHOD:**

We performed a discrete choice experiment with 55 international rifampicin-resistant tuberculosis physicians. We employed mixed-effects multinomial logistic regression to quantify the effect of prBDQ^R^, patient attributes, and contextual factors on the decision to continue BDQ or not when sequencing results become available.

**RESULTS:**

PrBDQ^R^ was the most influential factor for BDQ decision-making, three times greater than treatment response. Each percentage point increase in prBDQ^R^ resulted in 8.2% lower odds (OR 0.92, 95% CI 0.90–0.93) of continuing BDQ as a fully effective drug and 5.0% lower odds (OR 0.95, 95% CI 0.94–0.96) of continuing it but not counting it as an effective drug. The most favourable patient profile for prescribing BDQ as a fully effective drug was a patient receiving the BPaLM regimen (BDQ, pretomanid, linezolid and moxifloxacin) with low prBDQ^R^, good 1-month treatment response, fluoroquinolone-susceptible TB, and no prior BDQ treatment. Physicians with higher discomfort with uncertainty and more years of experience with BDQ were more inclined to stop BDQ.

**CONCLUSION:**

Given the uncertainty of genotype-phenotype associations, physicians valued prBDQ^R^ for BDQ decision-making in rifampicin-resistant TB treatment.

## RÉSUMÉ

**CONTEXTE:**

Le diagnostic précis de la résistance à la bédaquiline (BDQ) reste difficile. Une approche bayésienne exprime cette incertitude sous la forme d'une probabilité de résistance au BDQ (prBDQ^R^) avec un intervalle de crédibilité à 95%. Nous avons étudié comment les informations prBDQ^R^ influencent les décisions de prescription de BDQ.

**MÉTHODE:**

Nous avons réalisé une expérience à choix discret auprès de 55 médecins internationaux spécialisés dans la TB résistante à la rifampicine. Nous avons utilisé une régression logistique multinomiale à effets mixtes pour quantifier l'effet du prBDQ^R^, des attributs du patient et des facteurs contextuels sur la décision de poursuivre ou non le BDQ lorsque les résultats du séquençage sont disponibles.

**RÉSULTATS:**

La prBDQ^R^ a été le facteur le plus influent dans la prise de décision en matière de BDQ, trois fois plus importante que la réponse au traitement. Chaque augmentation d'un point de pourcentage du prBDQ^R^ a entraîné une baisse de 8,2% des probabilités (RC 0,92 ; IC à 95% 0,90–0,93) de poursuivre le BDQ en tant que médicament pleinement efficace et une baisse de 5,0% des probabilités (RC 0,95 ; IC à 95% 0,94–0,96) de le poursuivre mais de ne pas le compter comme un médicament efficace. Le profil de patient le plus favorable à la prescription de BDQ en tant que médicament pleinement efficace était celui d'un patient recevant le régime BPaLM (BDQ, pretomanid, linezolid et moxifloxacin) avec un faible prBDQ^R^, une bonne réponse au traitement à 1 mois, une tuberculose sensible aux fluoroquinolones et aucun traitement antérieur par BDQ. Les médecins présentant un plus grand malaise face à l'incertitude et plus d'années d'expérience avec la BDQ étaient plus enclins à arrêter la BDQ.

**CONCLUSION:**

Compte tenu de l'incertitude des associations génotype-phénotype, les médecins ont valorisé prBDQ^R^ pour la prise de décision BDQ dans le traitement de la TB RR.

Bedaquiline (BDQ) has become a key drug for treating rifampicin-resistant TB (RR-TB). Unfortunately, BDQ drug susceptibility testing (DST) does not keep pace with its widespread use. The development of a rapid genotypic DST is hindered by an incomplete understanding of the genomic basis of BDQ resistance, with many *Rv0678* variants being of ‘uncertain significance.’^1^ This uncertainty poses challenges for physicians in interpreting test results.

A Bayesian approach to predict the probability of BDQ resistance (prBDQ^R^) from genotypic data was recently proposed as an alternative solution.^2^ The prBDQ^R^ estimates the likelihood of BDQ resistance for a specific Rv0678 variant, with a 95% credible interval indicating the range of the true prBDQ^R^ value. Accompanying the report of an Rv0678 variant with its prBDQ^R^ could assist physicians in their decision-making. This probabilistic approach is, however, new to physicians who are used to binary DST results. To explore the clinical application of prBDQ^R^, we quantified the influence of prBDQ^R^ information on physicians’ judgement of the clinical value of BDQ for specific patients by investigating decisions to prescribe BDQ.

## METHODS

### Study design

A discrete choice experiment (DCE) was conducted to quantify the relative importance of prBDQ^R^ and other factors in physicians’ decisions to continue BDQ in the treatment regimen after receiving next-generation sequencing (NGS) results.

To design and implement the DCE survey, we followed a systematic and multi-step process guided by the recommendations of the International Society for Pharmacoeconomics and Outcomes Research and recently published state-of-the-art DCE studies for decision-making in healthcare.^3–5^

### Step 1: Selection of DCE attributes and levels

We started from a comprehensive list of attributes generated by a qualitative study that explored factors influencing physicians’ decision-making regarding the use of BDQ.^6^ The final selection of attributes was discussed by an expert panel using the following criteria for DCE factors: 1) influencing the decision to prescribe BDQ, 2) being ‘tradeable’ by not having an extreme impact on decision-making and 3) being independent (not correlated with) other attributes.^4,7^ The attribute levels were chosen to ensure clinical plausibility and ‘tradeability’ (i.e., not dominating decision-making).^7,8^ The expert panel agreed on a final set of six patient attributes and their corresponding levels (Table 1), five physician characteristics and three setting characteristics.

**Table 1.** DCE attributes and their levels.

	Attribute	Definition	Level		
1	Response to treatment at 1-month follow-up	The clinical symptoms and microbiology test results indicate the patient’s response to RR-TB treatment after the first month of treatment. The 1-month timepoint is chosen as it is the approximate time when NGS results are available	Good response: cough less, gaining weight, smear negative, culture pending		No improvement: still coughing, weight unchanged, smear +1, culture pending
2	Resistance profile	The resistance profiles of the infecting Mtb strain, as determined by the NGS performed on baseline Mtb culture	MDR-TB	MDR-TB + PZA + EMB resistance	MDR-TB + fluoroquinolones resistance
3	BDQ exposure history	Whether the patient was previously treated with a BDQ-containing regimen for TB	No prior exposure to BDQ		Treated for RR-TB with BDQ
4	BDQ resistance probability	The probability of BDQ resistance for a specific variant is the most likely estimate of the probability that an isolate containing that variant will be resistant to BDQ	20%	45%	70%
5	Credible interval of BDQ resistance probability	The 95% credible interval of the BDQ resistance probability, which expresses the uncertainty around this estimate and gives the range in which the true value lies	Narrow (±10%)		Wide (±20%)
6	Initial RR-TB regimen	The regimen that the patient initiated before obtaining NGS results	9-month regimen: BDQ+LFX+LZD+EMB + high-dose INH +PZA +CFZ		BPaLM regimen: BDQ, pretomanid, LZD, moxifloxacin

RR-TB = rifampicin-resistant TB; NGS = next-generation sequencing; Mtb = *M. tuberculosis*; MDR-TB = multidrug-resistant TB; BDQ = bedaquiline; LFX = levofloxacin; LZD = linezolid; EMB = ethambutol; INH = isoniazid; PZA = pyrazinamide; CFZ = clofazimine.

### Step 2: Experimental design and survey piloting

The DCE was designed as a single-profile, three-category-response choice experiment in which physicians were presented with hypothetical patients using narrative vignettes (Supplementary Data 1). We employed the statistical software package JMP Pro 17 (SAS Institute, Cary, NC, USA) to generate a fractional factorial experimental design with 42 unique patient profiles,^9^ partitioned into three blocks of 14 profiles administered to different participants to reduce the burden on them. All patients described in the vignettes had initiated a BDQ-containing ambulatory regimen for RR-TB before the NGS results were available. Clinical information not part of the six patient attributes was included as fixed information in all vignettes. At the 1-month follow-up visit, the approximate time when NGS results become available, physicians were asked for each hypothetical patient whether they would 1) continue BDQ as a fully effective drug, 2) continue BDQ but not count it as a fully effective drug, or 3) stop BDQ, and to indicate the certainty of their answers on a scale of 10. To assess reliability, one survey vignette was presented twice. The survey was pilot-tested with four physicians.

### Step 3: Data collection

Physicians with experience in managing RR-TB patients and directly involved in prescribing RR-TB treatment were eligible to participate in the DCE study. Recruitment occurred through invitations to participants and alumni of the course “Clinical Decision-making for Drug-resistant Tuberculosis”, organised by the Institute of Tropical Medicine (Antwerp, Belgium)^10^, invitations to RR-TB physicians identified by the study investigators, and snowball sampling. The DCE was performed online using the Qualtrics Survey platform. Following informed consent, participants were asked about their characteristics and randomly assigned to one of the three blocks of vignettes.

### Sample size

After recruiting the first 25 participants, we performed an interim analysis to simulate the number of participants required to have 80% power to detect the effect of prBDQ^R^ at the overall significance level of 5%.^11,12^ Based on these results, a final sample size of at least 42 participants was required to detect the effect of a high (≥70%) level of prBDQ^R^. (Supplementary Data 2).

#### Data analysis

To determine the relative importance of each attribute and level in the BDQ prescribing decision-making process, we modelled the three-category outcome (stop BDQ, continue BDQ but not count it as a fully effective drug, or continue BDQ as a fully effective drug) as a function of the attribute levels using a mixed-effects multinomial logistic model. A three-level outcome was chosen based on the results of a qualitative study.^6^ We started with the full model for which the design was constructed, involving all attribute main effects and the interaction terms between prBDQ^R^ and resistance profile, ambulatory regimen, BDQ exposure history and credible interval of prBDQ^R^. Important interaction terms were chosen by backward selection from the full model using Akaike’s information criterion (AIC).

Likelihood ratio tests were used to quantify each attribute's overall significance. We expressed each attribute's relative importance by the logworth statistic, i.e., log_10_(*P*-value of the likelihood ratio test).^13^ We explored the influence of physician and setting characteristics by adding those variables and their interaction terms with prBDQ^R^ to the regression model. The interaction terms remained in the model if the model’s AIC was lower than the model with only main effects.

Intra-class correlation was calculated to estimate the proportion of the variance explained by the preference heterogeneity among participants.^14^ The content validity of the DCE was assessed by comparing the sign of the estimated model parameter with the *a priori* hypothesis for each attribute.^15^ Measurement reliability was assessed by test-retest stability for the vignette that was presented twice.^15^ A sensitivity analysis was conducted to assess whether excluding choices with certainty lower than 4 points out of 10 affected the model's findings. The free-text comments of participants on each patient vignette and choice task were analysed qualitatively using a thematic approach to understand the reasoning behind physicians’ treatment decisions.

### Ethics approval and consent to participate

The study protocol was approved by the Research Ethics Committee of the University Hospital of the University of Antwerp, Antwerp, Belgium (Reference number 3395). All participants provided electronic informed consent before study participation.

## RESULTS

### Participant characteristics

Fifty-five participants were enrolled between 25 May 2022 and 16 May 2023. Participant characteristics are described in Table 2. Of the 48 participants who completed the test-retest exercise, 28 (58%) responded similarly to the repeated choice task. The DCE tasks were rated as quite difficult by 38% of the participants and very difficult by 14%.

**Table 2.** Characteristics of physicians participating in the DCE (*n* = 55).

Characteristic	(*n* = 55)
	*n* (%)
Participant setting	
Continent*	
Africa	21 (38.2)
Asia	18 (32.7)
Europe	8 (14.5)
America	2 (3.6)
Country income classification and burden of MDR-TB*	
HMIC – high MDR-TB burden	3 (5.5)
HMIC – low MDR-TB burden	12 (21.8)
LMIC – high MDR-TB burden	23 (41.8)
LMIC – low MDR-TB burden	11 (20.0)
Type of hospital*	
Non-academic hospital	24 (43.6)
Research/academic hospital	25 (45.5)
Proportion of eligible RR-TB patients in facility receiving BDQ, %, median [IQR]	100 [100–100]
Participant characteristic	
Age group, years*	
20–29	6 (10.9)
30–39	24 (43.6)
40–49	12 (21.8)
≥50	7 (12.7)
Years of experience in managing RR-TB, median [IQR]	6 [2–9]
Years of experience in using BDQ for RR-TB treatment, median [IQR]	3 [1–5]
Average number of initial RR-TB treatment decisions made per year^†^	
<5 patients	12 (21.8)
5–10 patients	5 (9.1)
11–20 patients	2 (3.6)
>20 patients	23 (41.8)
Percentage of BDQ-eligible RR-TB patients prescribed a BDQ-containing regimen, %^†^	90 [62.5–100]
Perception of BDQ: “I believe that the evidence is robust to prescribe a BDQ-containing regimen to all RR-TB patients who have no contraindication for BDQ”^†^	
Strongly disagree	2 (3.6)
Moderately disagree	0 (0.0)
Slightly disagree	5 (9.1)
Slightly agree	6 (10.9)
Moderately agree	7 (12.7)
Strongly agree	22 (40.0)
Risk-taking score, median [IQR]^†^	21 [17–23]
Reaction to uncertainty score, median [IQR]^†^	50 [46–54]

* Missing in 6 participants.

† Missing in 13 participants.

MDR-TB = multidrug-resistant TB; HMIC = high- and upper-middle-income country; LMIC = low- and lower-middle-income country; RR-TB = rifampicin-resistant TB; BDQ = bedaquiline; IQR = interquartile range.

### Effect of prBDQ^R^ and patient characteristics on physicians’ decision-making

The final model was a mixed-effects multinomial logistic model with a continuous prBDQ^R^ variable and included only main effects. The effects of prBDQ^R^, response to treatment at 1-month follow-up, a pre-extensively drug-resistant TB (pre-XDR-TB) resistance profile, and BDQ exposure history were statistically significant (Table 3). Notably, each percentage point increase in the prBDQ^R^ was associated with 5.0% lower odds (odds ratio [OR] 0.95, 95% confidence interval [CI] 0.93–0.96) of prescribing BDQ but not counting it as an effective drug and 8.2% lower odds (OR 0.92, 95% CI 0.90–0.93) of prescribing BDQ as a fully effective drug, compared to not prescribing BDQ. The odds of continuing BDQ (either as a fully effective drug or not) also significantly decreased if a patient had no improvement in treatment response at 1-month follow-up, had pre-XDR-TB, or had a prior history of exposure to BDQ. A narrow credible interval of prBDQ^R^ and the type of initial RR-TB regimen had no significant effect on physicians’ decision-making.

**Table 3.** Effect of patient characteristics on physicians’ decision-making about prescribing BDQ for RR-TB patients.

	Continue BDQ as a non-fully effective drug vs discontinue BDQ			Continue BDQ as a fully effective drug vs discontinue BDQ		
	Regression coefficient (SE)	OR (95%CI)	*P*-value	Regression coefficient (SE)	OR (95%CI)	*P*-value
BDQ resistance probability (effect of 1% increase)	–0.051 (0.007)	0.95 (0.94–0.96)	<0.001	–0.08 (0.008)	0.91 (0.90–0.93)	<0.001
Response to treatment at 1-month follow-up						
No improvement	Reference		<0.001	Reference		<0.001
Good response	1.078 (0.268)	2.94 (1.72–5.03)		2.028 (0.301)	7.60 (4.16–13.89)	
Resistance profile						
MDR-TB	Reference			Reference		
MDR-TB + PZA + EMB resistance	–0.414 (0.367)	0.66 (0.32–1.38)	0.26	–0.717 (0.411)	0.49 (0.21–1.11)	0.087
Pre-XDR-TB (RR-TB + FQ resistance)	–0.611 (0.302)	0.54 (0.30–0.99)	0.048	–1.387 (0.334)	0.25 (0.13–0.49)	<0.001
BDQ exposure history						
No prior exposure to BDQ	Reference		0.22	Reference		0.016
Treated for RR-TB with BDQ	–0.317 (0.253)	0.73 (0.44–1.21)		–0.694 (0.280)	0.50 (0.29–0.88)	
Credible interval of BDQ resistance probability						
Wide	Reference		0.74	Reference		0.76
Narrow	0.082 (0.250)	1.09 (0.66–1.79)		–0.083 (0.274)	0.92 (0.53–1.60)	
Ambulatory regimen						
9-month regimen	Reference		0.087	Reference		0.37
BPaLM regimen	–0.519 (0.297)	0.60 (0.33–1.08)		0.294 (0.331)	1.34 (0.69–2.60)	

BDQ = bedaquiline; RR-TB = rifampicin-resistant TB; SE = standard error; OR = odds ratio; CI = confidence interval; MDR-TB = multidrug-resistant TB; PZA = pyrazinamide; EMB = ethambutol; XDR-TB = extensively drug-resistant TB; FQ = fluoroquinolone; BPaLM = BDQ, pretomanid, linezolid, moxifloxacin.

The intra-class correlation was 0.37 for ‘prescribe BDQ as a fully effective drug’ vs ‘not prescribe BDQ’ and 0.43 for ‘prescribe BDQ but not as a fully effective drug’ vs ‘not prescribe BDQ,’ implying that physician heterogeneity could explain a moderate amount of variation in treatment decisions.

**Figure 1.**
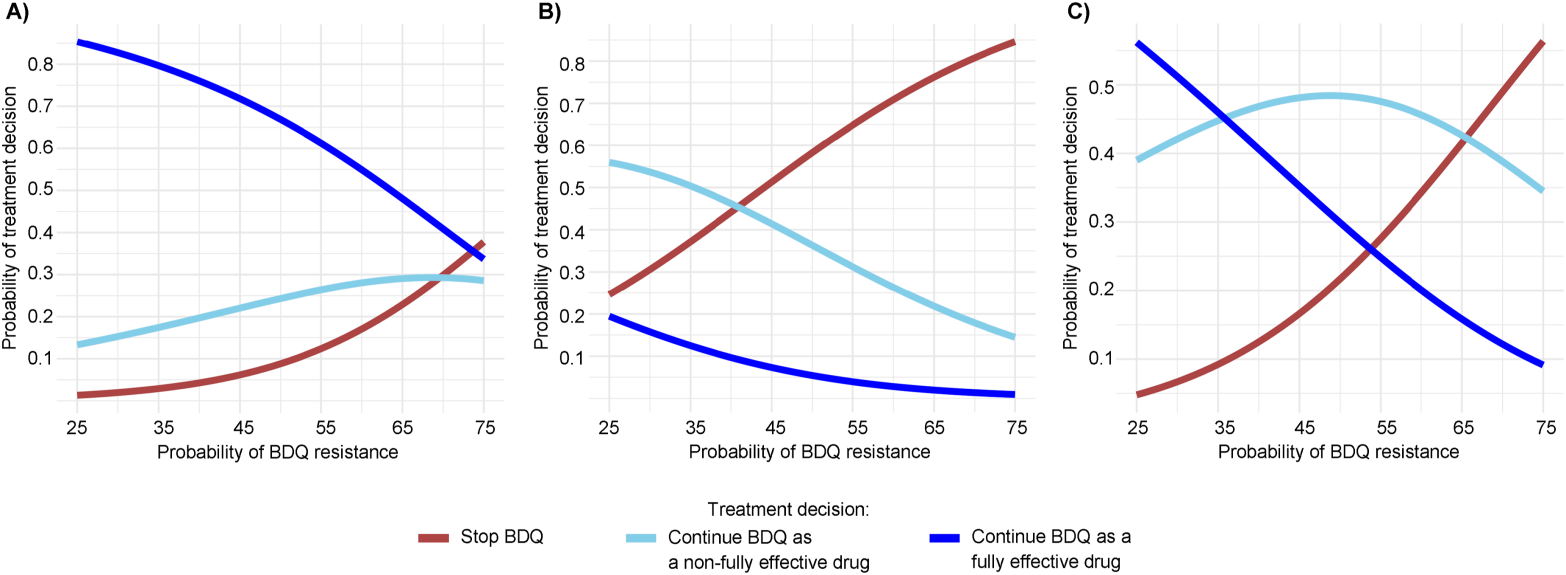
Predicted probability of treatment decision by prBDQ^R^ for **A)** a patient with MDR-TB, good treatment response, no history of exposure to BDQ, initiated on BPaLM regimen, and narrow credible intervals for prBDQ^R^; **B)** a patient with pre-XDR-TB, no improvement in treatment response, previously exposed to BDQ, initiated on the 9-month regimen, and narrow credible intervals for prBDQ^R^; **C)** a patient with MDR-TB + PZA + EMB resistance, good treatment response, previously exposed to BDQ, initiated on the 9-month regimen, and narrow credible intervals for prBDQ^R^. BDQ = bedaquiline; prBDQ^R^ = probability of BDQ resistance; BPaLM = BDQ, pretomanid, linezolid, moxifloxacin; MDR-TB = multidrug-resistant TB; PZA = pyrazinamide; EMB = ethambutol.

The effect estimates show that the most favourable patient profile for the decision to continue BDQ as a fully effective drug is a patient with good response to treatment at 1-month follow-up, *Mycobacterium tuberculosis* (Mtb) susceptible to fluoroquinolones (FQs), no history of prior exposure to BDQ, use of BPaLM (bedaquiline, pretomanid, linezolid and moxifloxacin) as the initial regimen, and a narrow credible interval of prBDQ^R^. Physicians only stopped BDQ for such patients when the prBDQ^R^ reached 75% (Figure 1A). In contrast, for patients with pre-XDR-TB who failed to improve clinically after one month of treatment and who were treated with BDQ during a previous RR-TB episode, physicians preferred to stop BDQ when the prBDQ^R^ reached 40% (Figure 1B). For a patient with a profile in between these two extremes, physicians were likely to continue BDQ as a fully effective drug if the PrBDQ^R^ was below 35% and stop BDQ when the prBDQ^R^ exceeded 65%. If the PrBDQ^R^ was between 35% and 65%, they preferred to continue BDQ but not as an effective drug (Figure 1C).

**Figure 2.**
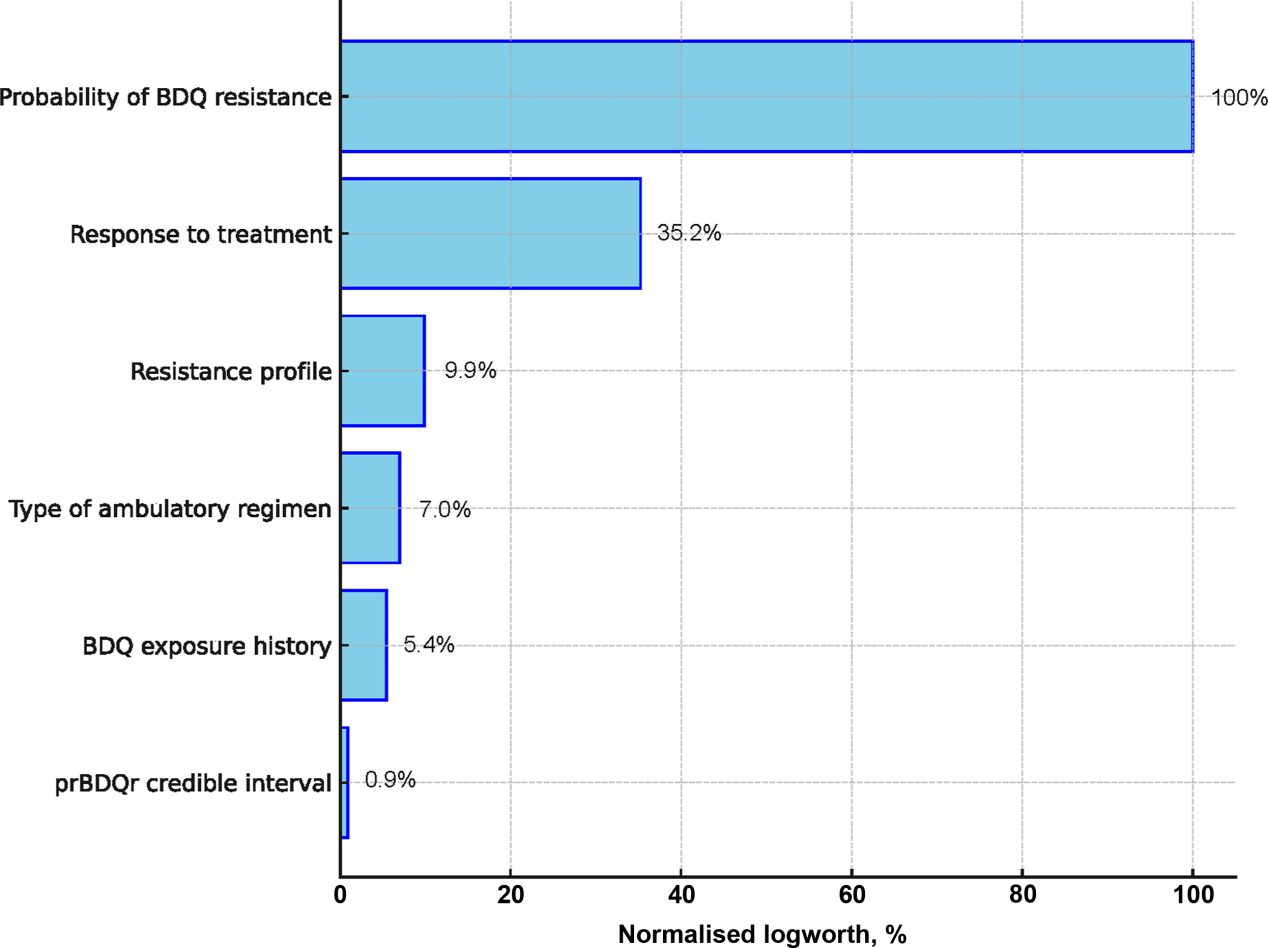
Relative importance of the six attributes compared to the most important attribute ‘probability of BDQ resistance’ (normalised logworth of 100%). BDQ = bedaquiline.

Figure 2 illustrates the importance of the different attributes on the logworth scale relative to the prBDQ^R^ attribute. The prBDQ^R^ was approximately three times more important than the treatment response and ten times more than the patient’s resistance profile. The attribute credible interval of prBDQ^R^ played the least important role in physicians’ decisions to continue or stop BDQ.

### Influence of physician and setting characteristics on treatment choice

Physician- and setting-related variables were added to the model to explore observed heterogeneity among physicians in their BDQ prescription decision, particularly differences in the perceived relative importance of prBDQ^R^ (Supplementary Data 3). Physicians with more years of experience in using BDQ (OR 0.62–0.71 for every year increase in BDQ experience) and those with higher discomfort with uncertainty (OR 0.86–0.89 for every 1-point increase in uncertainty score) were less likely to continue BDQ. Interactions between prBDQ^R^ and physician- and setting-related characteristics were not identified.

### Sensitivity analysis

There were 42 out of 653 choices made for which participants rated their certainty lower than or equal to 4 out of 10. Results were similar to those of the current model when these choices were excluded (Supplementary Data 4).

### Qualitative comments

Physicians remarked that they opted to continue BDQ but not as an effective drug when there was a discordance between patient response and prBDQ^R^, when prBDQ^R^ was low for a patient who was previously treated with a BDQ-containing regimen, or when the patient’s Mtb strain was FQ-resistant. Some physicians found it challenging to use prBDQ^R^ in their treatment decisions because they were not familiar with employing NGS results in patient management or had limited experience with managing BDQ-resistant TB. Several physicians expressed uncertainty about the inference of BDQ phenotype from genotypic results. The full results of the qualitative analysis and accompanying quotes are provided in Supplementary Data 5.

## DISCUSSION

We performed a DCE to quantify the effect of the prBDQ^R^ on physicians’ decision-making for RR-TB management. The prBDQ^R^ was the most significant factor influencing physicians’ decisions to prescribe BDQ. The most favourable patient profile for prescribing BDQ as a fully effective drug when sequencing results become available is a patient with a low prBDQ^R^, a good response after 1 month of treatment, FQ-susceptible TB, no prior BDQ use, and receiving BPaLM.

Studies of medical decision-making showed that sources of uncertainty include the indeterminacy of the treatment outcome, and ambiguity and complexity of risk information.^16^ In the context of BDQ decision-making once genomic DST results are available, uncertainty arises from the many factors affecting RR-TB treatment response, challenges in inferring BDQ phenotype from genotype, and the limited understanding of the effect of the presence of a variant in the *Rv0678* gene on RR-TB treatment outcomes. Until the impact of the presence of a specific genomic variant in Mtb on the effectiveness of BDQ for a particular patient can be determined with sufficient certainty, communicating prBDQ^R^ values could be a helpful approach to predict the clinical usefulness of BDQ for individual patients and promote judicious BDQ use.

Decision-making under uncertainty also depends on physicians’ personal traits, such as risk attitudes and cognitive biases.^17–19^ We observed that physicians who reported high discomfort with uncertainty were more likely to modify or intensify the treatment regimen than those with lower discomfort. This is consistent with previous findings that physicians may favour action over inaction in instances of high uncertainty^20^ and that physicians’ low tolerance for uncertainty may lead to ordering more tests and offering interventions.^21^

Our study confirmed two key findings of a previous qualitative study. Firstly, when making treatment decisions, prBDQ^R^ should not be viewed as stand-alone information but should be interpreted in the context of patient characteristics. Decision thresholds for prBDQ^R^ are not fixed, but vary with changing patient characteristics.^6^ For example, the DCE model predicts that at a 50% prBDQ^R^, physicians would continue BDQ as a fully effective drug if the patient had a favourable profile, but would stop BDQ if the patient had pre-XDR-TB and did not improve clinically after 1 month of treatment and had previously been treated with BDQ. Secondly, continuing BDQ but not counting it as a fully effective drug by strengthening the regimen is a valid option when the patient has risk factors for both good and poor treatment outcomes. This approach maximises the likelihood that a patient will benefit from receiving BDQ in the event that BDQ is still effective and minimises the risk of amplifying resistance if the Mtb strain were to be resistant to BDQ, which would result in fewer than three effective drugs in the BPaLM treatment regimen.^22^ The option to strengthen the regimen further highlights the importance of using NGS to obtain a comprehensive resistance profile, which is needed to design an evidence-based, robust treatment regimen.^23^

This study must also be interpreted considering its limitations. First, we could only investigate a limited number of attributes and individual traits, while in reality, the range of factors physicians consider when making decisions may be much more complex. Second, the test-retest reliability of the DCE was only moderate, indicating that the DCE tasks may be too complicated for some participants and that there may be a learning curve effect.^24,25^ Third, the convenience sample may have induced selection bias. Fourth, the prBDQ^R^ of a specific variant in *Rv0678* must be interpreted with information on *mmpL5*, as epistatic interactions can occur.^26^ Fifth, numerous challenges must be overcome before prBDQ^R^ can be implemented in clinical care. Frequent updates are required to accurately estimate prBDQ^R^ for individual genomic variants as new evidence emerges. Finally, genome sequencing is needed, which requires considerable efforts to build laboratory and bioinformatics capacity in high RR-TB burden countries.^27,28^

## CONCLUSIONS

The novel concept of prBDQ^R^ helps communicate the consequence of a specific *Rv0678* gene variant to physicians. The decision thresholds for prBDQ^R^ are not fixed, but depend on patient characteristics. Physicians often opted to continue BDQ and strengthen the regimen when the prBDQ^R^, in combination with patient characteristics, suggested uncertainty about its clinical value. Future studies should investigate whether applying the prBDQ^R^ in practice promotes the optimal use of BDQ and improves treatment outcomes.

## Supplementary Material


