# Supplementary material for: Impact of bedaquiline resistance probability on treatment decision for rifampicin-resistant TB
Source: IJTLD Open. 2024 Sep 1;1(9):384–90. doi: 10.5588/ijtldopen.24.0362 (PMC11409166; doi:10.5588/ijtldopen.24.0362)
Supplement: Supplementary file 1 [file ijtldopen24-0362_supplementarydata1.pdf]

http://dx.doi.org/10.5588/ijtldopen.24.0362

Impact of bedaquiline resistance probability on treatment decision for rifampicin-resistant TB

Supplementary Data 1. Example of a patient vignette

A 48-year-old woman was diagnosed with RR-TB in 2019. She was treated with all-oral BDQ-containing RR-TB regimen and successfully completed her prescribed regimen.

In March 2022, she was diagnosed with RR-TB on Xpert. At diagnosis, her BMI was 20.8 kg/m^2. Smear microscopy was positive (2+). The patient was started on the WHO-approved 6-month BPaLM regimen consisting of BDQ + Pretomanid + LZD + MFX.

At the 1-month routine follow-up visit, the patient has responded well to treatment, coughing less and gaining weight. She reports good adherence. She does not have any side effect of TB drugs.

One week later, the following results become available:

- Smear microscopy of the sputum sample collected at month 1 of treatment: negative
- Culture of sputum collected at the month 1 visit: result pending
- WGS performed on baseline Mtb culture:
    - Resistant to RIF and INH
    - BDQ: 337G>A variant is detected in the *Rv0678* gene. The estimated probability of BDQ resistance is 45% with wide credible interval of 25% - 65%
    - Susceptible to PZA, EMB, LFX, MFX, ETH, SM, AMK, CFZ, LZD, DLM, CYCLO, TRD, PAS

Do you (in your setting) continue BDQ for this patient?

☐ 1. Yes, I continue BDQ and count it as a fully active drug

☐ 2. Yes, I continue BDQ but I strengthen the regimen by adding one or more drugs because I do not count BDQ as an active drug

☐ 3. No, I stop BDQ and design another regimen

Please rate on a scale from 1 to 10 how certain you are with this decision:

Very uncertain 1 2 3 4 5 6 7 8 9 10 Very certain

If you chose option 2:

☐ List drug(s) you would add to the regimen:__________

☐ List drug(s) you would stop: __________

☐ I will defer decision to the provincial/regional/national expert committee

If you chose option 3:

☐ Which regimen would you prescribe: __________

☐ I will defer decision to the provincial/regional/national expert committee

Do you have other comments on this patient?

Supplementary Data 2. Simulation for sample size estimation

1. Methods

After recruiting the first 25 participants, we performed an interim analysis to simulate the number of participants required to have 80% power to detect the effect of prBDQ[R] at the overall significance level of 5%.

The data from the first 25 participants were analysed using the random-effect proportional odds model. The model included a random intercept for participant ID, main effects of six patient attributes, and four interaction effects between resistance probability and ambulatory regimen, resistance profile, exposure history and credible interval. All attributes were specified as categorical variables.

The parameter estimates from the proportional odds model served as input to the simulation. The model was specified as follows:

$$logit\big(P(y_{ik} \le j)\big) = \mu_j - \beta_1 * treatment\ response_i - \beta_2 * resistance\ profile\ PZA_i - \beta_3 * resistance\ profile\ FQ_i - \beta_4 * ambulatory\ regimen - \beta_5 * exposure\ history_i - \beta_6 * probability\ 45_i - \beta_7 * probability\ 70_i - \beta_8 * credible\ interval_i - \beta_9 * prob_{45}{:}PZA_i - \beta_{10} * prob_{45}{:}FQ_i - \beta_{11} * prob_{70}{:}PZA_i - \beta_{12} * prob_{70}{:}FQ_i - \beta_{13} * prob_{45}{:}ambu\ regimen_i - \beta_{14} * prob_{70}{:}ambu\ regimen_i - \beta_{15} * prob_{45}{:}exposure\ history_i - \beta_{16} * prob_{70}{:}expo\ history_i - \beta_{17} * prob_{45}{:}cred\ interval_i - \beta_{18} * prob_{70}{:}cred\ inteval_i - u_k * physician_k$$

with:

y_i: categorical outcome of profile *i* answered by physician *k*, with *J* = 3 categories

j: an outcome category, *j* = 1,..., *J*-1

μ_j: intercept for category *j*; $\mu_1 = -4.023, \mu_2 = -1.773$

u_k: physician-level random effect, $u_k \sim N(0, 2.37)$

$\beta_1, \dots, \beta_{18}$: model parameters

$\beta_1 = -1.667, \beta_2 = -0.676, \beta_3 = -1.575, \beta_4 = 1.121, \beta_5 = 0.175, \beta_6 = -1.781, \beta_7 = -2.916, \beta_8 = -1.237, \beta_9 = 0.692, \beta_{10} = 0.436, \beta_{11} = 0.136, \beta_{12} = 1.356, \beta_{13} = 0.138, \beta_{14} = -0.995, \beta_{15} = -0.858, \beta_{16} = -1.495, \beta_{17} = -0.996, \beta_{18} = 1.96$

We simulated 1000 datasets with sample sizes ranging from 30 to 100 with increments of 10. For each simulated dataset, we fitted a proportional odds model. We then calculated the power to detect each main effect in the model at the 5% significance level, adjusted for multiple testing using the Holm-Bonferroni method.

2. Results

The simulation results are displayed in Figure 1. For a sample size of 42 participants, we have at least 80% power to detect the effect of the “70%” level of prBDQ[R], as well as the “MDR-TB + fluoroquinolones resistance” level of the “resistance profile” attribute, and the “treatment response”, “ambulatory regimen” and “credible interval” attributes.

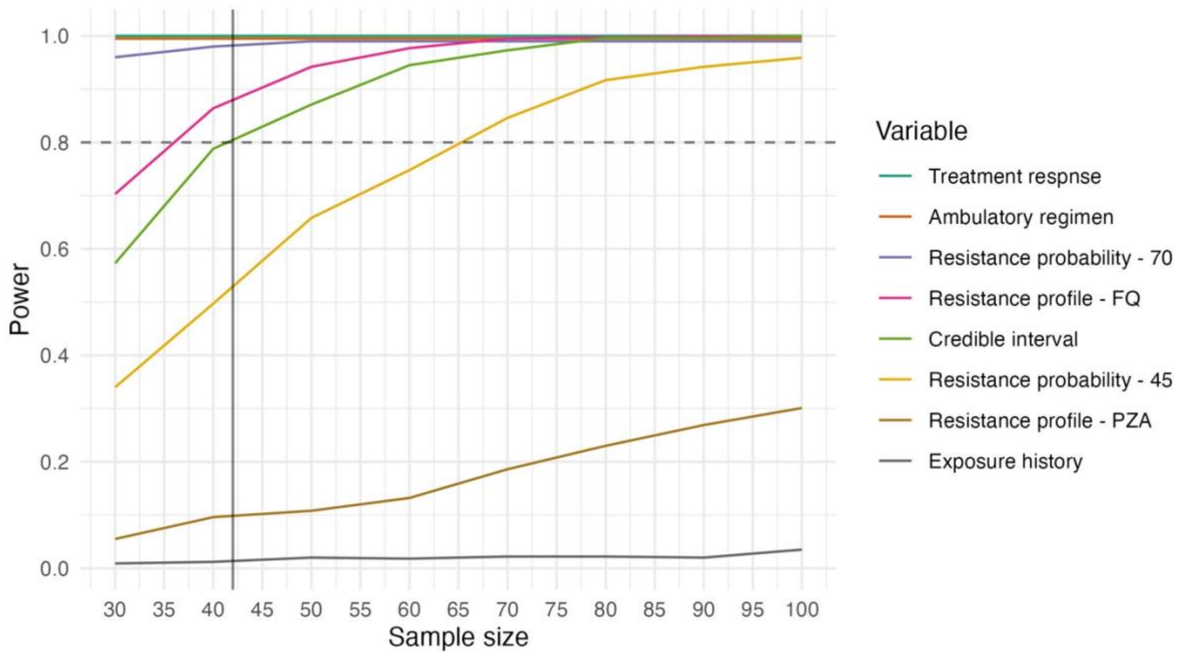

Figure 1. Power to detect an attribute level's main effect by sample size

Supplementary Data 3. Results of the mixed multinomial logit regression model with additional physician- and setting-related variables

		**Continue BDQ but not as a fully effective drug** versus **Stop BDQ**			**Continue BDQ as a fully effective drug** versus **Stop BDQ**		
**Term**		**Regression coefficient (Standard error)**	**OR (95% CI)**	**p-value**	**Regression coefficient (Standard error)**	**OR (95% CI)**	**p-value**
**BDQ resistance probability (effect of 1% increase in probability)**		-0.047 (0.007)	0.954 (0.940 - 0.969)	<0.001	-0.08 (0.008)	0.924 (0.908 - 0.94)	<0.001
**Response to treatment at 1-month follow up**							
	No improvement	Reference	-	<0.001	Reference	-	<0.001
	Good response	1.157 (0.285)	3.18 (1.789 - 5.654)		2.271 (0.318)	9.686 (5.085 - 18.448)	
**Resistance profile**							
	MDR-TB	Reference	-		Reference	-	
	MDR-TB + PZA + EMB resistance	-0.473 (0.385)	0.623 (0.286 - 1.357)	0.23	-0.612 (0.425)	0.542 (0.229 - 1.282)	0.158
	Pre XDR TB (RR-TB + FQ resistance)	-0.582 (0.318)	0.559 (0.294 – 1.063)	0.075	-1.358 (0.350)	0.257 (0.127 - 0.521)	<0.001
**BDQ exposure history**							
	No prior exposure to BDQ	Reference	-	0.173	Reference	-	0.021
	Treated for RR-TB with BDQ	-0.368 (0.266)	0.692 (0.404 - 1.184)		-0.698 (0.290)	0.497 (0.277 - 0.894)	
**Credible interval of BDQ resistance probability**							
	Wide	Reference	-	0.86	Reference	-	0.79
	Narrow	0.048 (0.263)	1.049 (0.616 - 1.787)		-0.075 (0.286)	0.927 (0.520 - 1.654)	
**Ambulatory regimen**							

	9-month all-oral regimen	Reference	-	0.089		Reference	-	0.25
	BPaLM regimen	-0.551 (0.316)	0.577<br>(0.394 - 1.092)			0.406 (0.350)	1.500<br>(0.739 – 3.045)	
**Physician's age group**								
	20-29 years old	Reference	-	-		Reference	-	-
	30-39 years old	-0.971 (0.995)	0.379<br>(0.051 – 2.832)	0.33		-3.061 (1.059)	0.047<br>(0.005 – 0.399)	0.006
	40-49 years old	-0.398 (0.948)	0.671<br>(0.099 – 4.571)	0.68		-2.627 (1.016)	0.072<br>(0.009 – 0.565)	0.013
	50 years old and above	1.724 1.292	5.608<br>(0.411 -76.594)	0.190		0.458 (1.380)	1.581<br>(0.097 - 25.789)	0.74
**Years of experience with BDQ use**		-0.343 (0.136)	0.710<br>(0.539 - 0.935)	0.016		-0.470 (0.148)	0.625<br>(0.463 - 0.844)	0.003
**Average number of RR-TB initial treatment decisions made per year**								
	< 5 patients	Reference	-	-		Reference	-	-
	5 – 20 patients	-0.513 (0.902)	0.599<br>(0.097 - 3.712)	0.57		0.886 (0.935)	2.425<br>(0.366 - 16.066)	0.35
	> 20 patients	0.457 (0.763)	1.579<br>(0.337 - 7.397)	0.55		0.918 (0.822)	2.506<br>(0.475 - 13.215)	0.27
**Risk-taking score (effect of 1 point increase)**		-0.1371 (0.075)	0.872<br>(0.749 - 1.014)	0.075		-0.118 (0.076)	0.888<br>(0.762 - 1.035)	0.126
**Uncertainty score (effect of 1 point increase)**		-0.153 (0.035)	0.858<br>(0.799 - 0.922)	<0.001		-0.118 (0.037)	0.888<br>(0.824 - 0.958)	0.037
**Perception on importance of BDQ in RR-TB treatment** (agreement with the statement "*I believe that the evidence is robust to prescribe a BDQ-containing regimen to all RR-TB patients who have no contraindication for BDQ*".)								
	Disagree*	Reference	-	-		Reference	-	-
	Slightly agree	-0.964 (0.997)	0.381<br>(0.051 - 2.865)	0.33		-2.797 (1.043)	0.061<br>(0.007 - 0.502)	0.011

Moderately agree	-2.537 (0.958)	0.079 (0.011 - 0.549)	0.012		-1.576 (0.955)	0.207 (0.03 - 1.426)	0.107
Strongly agree	-1.269 (0.708)	0.281 (0.067 - 1.178)	0.081		-2.843 (0.770)	0.058 (0.012 - 0.276)	<0.001
**Type of hospital**							
Academic/research hospital	Reference	-	-		Reference	-	-
Non-academic, non-research hospital	0.884 (0.555)	2.42 (0.788 - 7.434)	0.119		-0.194 (0.570)	0.824 (0.26 - 2.608)	0.73
**Country income classification and burden of MDR-TB**							
HMIC – high MDR-TB burden	-0.442 (1.257)	0.643 (0.051 - 8.167)	0.73		0.874 (1.350)	2.396 (0.156 - 36.782)	0.521
LMIC – high MDR-TB burden	0.258 (0.742)	1.295 (0.288 - 5.813)	0.73		0.535 (0.809)	1.708 (0.333 - 8.768)	0.511
LMIC – low MDR-TB burden	0.056 (0.999)	1.058 (0.14 7.98)	0.96		3.655 (1.051)	38.665 (4.61 - 324.266)	<0.001

** The categories “strongly disagree”, “moderately disagree”, “slightly disagree” were combined into one category because responses were sparse.*
BDQ = bedaquiline; RR-TB = rifampicin-resistant TB; SE = standard error; OR = odds ratio; CI = confidence interval; MDR-TB = multidrug-resistant TB; PZA = pyrazinamide; EMB = ethambutol; XDR-TB = extensively drug-resistant TB; FQ = fluoroquinolone; BPaLM = BDQ, pretomanid, linezolid, moxifloxacin; HMIC = High- and upper-middle-income country; LMIC = Low- and lower-middle-income country.

Supplementary Data 4. Sensitivity analysis: excluding choices with certainty lower than or equal to 4 out of 10

		**Continue BDQ but not as a fully effective drug** versus **Stop BDQ**				**Continue BDQ as a fully effective drug** versus **Stop BDQ**		
**Term**		**Regression coefficient (Standard error)**	**OR (95% CI)**	**p-value**		**Regression coefficient (Standard error)**	**OR (95% CI)**	**p-value**
**BDQ resistance probability (effect of 1% increase in probability)**		-0.054 (0.008)	0.947 (0.933 - 0.962)	< 0.0001		-0.089 (0.008)	0.915 (0.899 - 0.932)	<0.0001
**Response to treatment at 1-month follow up**								
	No improvement	Reference	-	0.0004		Reference	-	<0.0001
	Good response	1.043 (0.279)	2.839 (1.626 – 4.958)			1.962 (0.314)	7.116 (30787 - 13.375)	
**Resistance profile**								
	MDR-TB	Reference	-			Reference	-	
	MDR-TB + PZA + EMB resistance	-0.425 (0.380)	0.654 (0.305 - 1.401)	0.268		-0.603 (0.427)	0.547 (0.232 - 1.289)	0.164
	Pre XDR TB (RR-TB + FQ resistance)	-0.641 (0.314)	0.524 (0.279 - 0.982)	0.044		-1.485 (0.354)	0.225 (0.111 - 0.460)	0.0001
**BDQ exposure history**								
	No prior exposure to BDQ	Reference	-	0.173		Reference	-	0.004
	Treated for RR-TB with BDQ	-0.366 (0.265)	0.694 (0.408 - 1.180)			-0.873 (0.296)	0.418 (0.231 - 0.756)	
**Credible interval of BDQ resistance probability**								
	Wide	Reference	-	0.878		Reference	-	0.430
	Narrow	0.040 (0.260)	1.041 (0.618 - 1.753)			-0.23 (0.289)	0.795 (0.445 - 1.418)	
**Ambulatory regimen**								
	9-month all-oral regimen	Reference	-	0.085		Reference	-	0.392
	BPaLM regimen	-0.539 (0.307)	0.583 (0.315 - 1.081)			0.300 (0.347)	1.349 (0.672 - 2.708)	

Supplementary Data 5: Qualitative comments of participants in the DCE survey

Regarding participants' explanation of their treatment decision, low $prBDQ^R$ and good treatment response were commonly mentioned as the reasons to continue BDQ in the regimen (and vice versa). However, several (n=5) physicians commented that 1 month is too early to judge treatment response. Thus, even if a patient does not respond well to treatment after 1 month, the physician would not change the regimen. Several participants also remarked that the discordance between patient response and $prBDQ^R$ was puzzling and urged them to continue BDQ as a non-fully effective drug and strengthen the regimen.

> *"Month 1 is too early to conclude a patient is not responding to treatment especially without bacteriological follow up tests. I will monitor the patient."*
>
> *(Participant ID 86)*
>
> *"…using the early response is not necessarily a good marker of final outcome - in those not responding need to think of other reasons and in those responding one may still need to strengthen."*
>
> *(Participant ID 58)*
>
> *"For this patient I will not stop BDQ as she is responding to treatment though there is a high likelihood of resistance but I would want to add amikacin to reduce the chances of treatment failure."*
>
> *(Participant ID 24)*

The strategy of strengthening the regimen was also chosen when $prBDQ^R$ was low for a patient who was previously treated with a BDQ-containing regimen or when the patient was infected with fluoroquinolones-resistant TB strains (mentioned by at least four participants).

> *"We are losing core drugs Moxi and BDQ is also at risk. That's why if I assume BDQ as fully active drugs and construct the regime , probably I might lose BDQ totally as well. This is the idea I am thinking about new regimen."*
>
> *(Participant ID 18)*

When interpreting the $prBDQ^R$, some physicians also took into account the type of mutation or the mutation gene. For example, they regarded the mutations in the *atpE* gene, or single nucleotide insertions as having low possibility of conferring resistance.

> *"Is the atpE gene clinically relevant despite the high probability of bedaquiline resistance? Will take the risk to continue ongoing treatment."*
>
> *(Participant ID 33)*
>
> *"The single nucleotide insertion (418_419insG) in the Rv0678 gene from the WGS on BDQ might not confirm resistance, other patient factors will be considered as reasons for poor patient improvement."*

(Participant ID 17)

Physicians also indicated that they were not accustomed to using next-generation sequencing (NGS) or whole genome sequencing (WGS) results in patient management, or to deal with patients who have BDQ-resistant strains. Furthermore, 10 physicians stated that the uncertainty of inference of BDQ phenotype from genotypic results is an important hurdle for treatment decision-making based on NGS results.

> *"Difficult because of lack of knowledge about Rv0678/pepQ/atpE mutations; is it really a fixed resistance conferring mutation? Or a transient one that can be overcome? Also patients have been reported with successful outcome despite baseline Rv0678 mutations..."*

(Participant ID 5)

> *"But in my working area like resource limited setting where DST for new and repurposed drugs could not be available, I've not much experience for BDQ resistant cases so that I've low confidence about construction and regimen choice.[...] I think I will need to solve BDQ resistance cases in my area soon."*

(Participant ID 13)

At least two participants commented that they faced difficulty as the ambulatory regimens in some patient vignettes were not in line with current treatment guidelines. Particularly, they would not start a BDQ-containing regimen in case of prior exposure to BDQ.

> *"Because sometimes I wouldn't start that regimen since the beginning (e.g. BPaL or STR in case of previous exposure to BDQ), so it's always complicated to stop medication when the patient is improving."*

(Participant ID 16)

Some participants remarked that they would need more information than what was provided in the vignette, such as comorbidity, extent of the TB, and level of FQ resistance, to make an informed decision. Participants also commonly commented that they would like to discuss the cases with other clinicians and the expert committee.

> *"I would like to know the level of resistance to FQ before deciding whether the regime should be changed or can be continued."*

(Participant ID 12)
